# Tubulosine selectively inhibits JAK3 signalling by binding to the ATP‐binding site of the kinase of JAK3

**DOI:** 10.1111/jcmm.15362

**Published:** 2020-06-17

**Authors:** Byung‐Hak Kim, Eun Hee Yi, Jun‐Goo Jee, Ae Jin Jeong, Claudio Sandoval, In‐Chul Park, Gyeong Hun Baeg, Sang‐Kyu Ye

**Affiliations:** ^1^ Department of Pediatrics New York Medical College Valhalla NY USA; ^2^ Department of Pharmacology Seoul National University College of Medicine Seoul Republic of Korea; ^3^ Biomedical Science Project (BK21^PLUS^) Seoul National University College of Medicine Seoul Republic of Korea; ^4^ Ischemic/Hypoxic Disease Institute Seoul National University College of Medicine Seoul Republic of Korea; ^5^ Research Institute of Pharmaceutical Researches College of Pharmacy Kyungpook National University Daegu Republic of Korea; ^6^ Division of Basic Radiation Bioscience Korea Institute of Radiological and Medical Sciences Seoul Korea; ^7^ School of Life and Health Sciences Chinese University of Hong Kong Shenzhen China; ^8^ Neuro‐Immune Information Storage Network Research Center Seoul National University College of Medicine Seoul Republic of Korea

**Keywords:** JAK3, leukaemia, lymphoma, STAT, structure‐based computational database screening, tubulosine

## Abstract

Gain‐ or loss‐of‐function mutations in Janus kinase 3 (JAK3) contribute to the pathogenesis of various haematopoietic malignancies and immune disorders, suggesting that aberrant JAK3 signalling is an attractive therapeutic target to treat these disorders. In this study, we performed structure‐based computational database screening using the 3D structure of the JAK3 kinase domain and the National Cancer Institute diversity set and identified tubulosine as a novel JAK3 inhibitor. Tubulosine directly blocked the catalytic activity of JAK3 by selective interacting with the JAK3 kinase domain. Consistently, tubulosine potently inhibited persistently activated and interleukin‐2‐dependent JAK3, and JAK3‐mediated downstream targets. Importantly, it did not affect the activity of other JAK family members, particularly prolactin‐induced JAK2/signal transducer and activator of transcription 5 and interferon alpha‐induced JAK1‐TYK2/STAT1. Tubulosine specifically decreased survival and proliferation of cancer cells, in which persistently active JAK3 is expressed, by inducing apoptotic and necrotic/autophagic cell death without affecting other oncogenic signalling. Collectively, tubulosine is a potential small‐molecule compound that selectively inhibits JAK3 activity, suggesting that it may serve as a promising therapeutic candidate for treating disorders caused by aberrant activation of JAK3 signalling.

## 1 INTRODUCTION

Cytokines are critical signalling molecules that regulate various biological responses, including cell proliferation, differentiation, tissue homeostasis, haematopoiesis and immune responses. However, aberrant cytokine signalling can result in a wide variety of human diseases such as cancer and immune disorders.^1^ The Janus family of protein tyrosine kinases (JAKs) and their substrates (signal transducer and activator of transcription [STAT] proteins) are examples of important cytokine‐mediated downstream effectors to regulate these biological processes.^2^ The mammalian JAK family consists of four members: JAK1, JAK2, Janus kinase 3 (JAK3) and TYK2, and the STAT substrate family consists of seven members: STAT1, STAT2, STAT3, STAT4, STAT5A, STAT5B and STAT6.^2^ In particular, JAK3 is predominantly expressed in haematopoietic cell lineages such as lymphoid cells and mediates receptor‐mediated cytokine signalling involving interleukin (IL)‐2, IL‐4, IL‐7, IL‐9, IL‐15 and IL‐21.^3^ These cytokines transduce JAK3/STAT signalling cascades through the common gamma chain (γc) subfamily of cytokine receptors paired with an alpha or beta chain as signalling partners.^2^, ^3^ They also thought to be particularly involved in T‐cell development and immune homeostasis, as loss‐of‐function *JAK3* mutations in humans have been shown to result in haematopoietic disorders such as severe combined immunodeficiency (SCID).^4^, ^5^ Further, gene therapy for autosomal recessive SCID using haematopoietic stem cell transplantation increased the risk of acute T‐cell leukaemia due to the direct activation of the γc‐mediated JAK3/signal transducer and activator of transcription 5 (STAT5) signalling.^6^

Aberrantly activated JAK3/STAT signalling has been implicated in various haematologic cancers. For example, in leukaemic blast cells, JAK3/STAT signalling was persistently activated in 70% of patients with acute myeloid leukaemia (AML).^7^ It was also observed in various haematologic cancer cell lines, including anaplastic large cell lymphoma,^8^ Burkitt's lymphoma,^9^ mantle‐cell lymphoma^10^ and enteropathy‐associated T‐cell lymphoma.^11^ In addition, constitutively active JAK3/STAT signalling is reported in the mouse model of pre‐B‐cell leukaemia. This model is established by loss‐of‐function mutations of the tumour suppressor B‐cell linker (BLNK), an inhibitor that binds JAK3 and decreases autocrine JAK3/STAT5 signalling.^12^ In this model, BLNK expression was completely lost or drastically reduced in paediatric pre‐B‐cell acute lymphoblastic leukaemia (ALL) cases.^13^ Somatic mutations of *JAK3* alleles have also been identified in cancer cell lines, as well as in patients with the following diseases: acute megakaryoblastic leukaemia,^14^, ^15^ high‐risk childhood ALL,^16^, ^17^ Down syndrome AML and ALL,^18^ T‐cell ALL^19^ and cutaneous T‐cell lymphomas.^20^ In these cases, the patients acquired constitutive‐active JAK3/STAT signalling by gain‐of‐function. This evidence suggests that aberrantly activated JAK3/STAT signalling contributes to the pathogenesis of a subset of haematopoietic malignancies and JAK3 is an attractive therapeutic target for the treatment of patients with these diseases.

In this study, we aimed to discover the small‐molecule inhibitors of JAK3 and identified tubulosine as a potent JAK3 inhibitor. Tubulosine potently inhibited constitutively active and IL‐2‐induced JAK3/STAT signalling, thereby decreasing proliferation and survival of cancer cells by inducing apoptotic and necrotic/autophagic cell death. These findings indicate that tubulosine may be a promising candidate for therapeutic intervention in diseases caused by abnormal JAK3 activity.

## 2 MATERIALS AND METHODS

### 2.1 Chemicals and reagents

**FIGURE 1:**
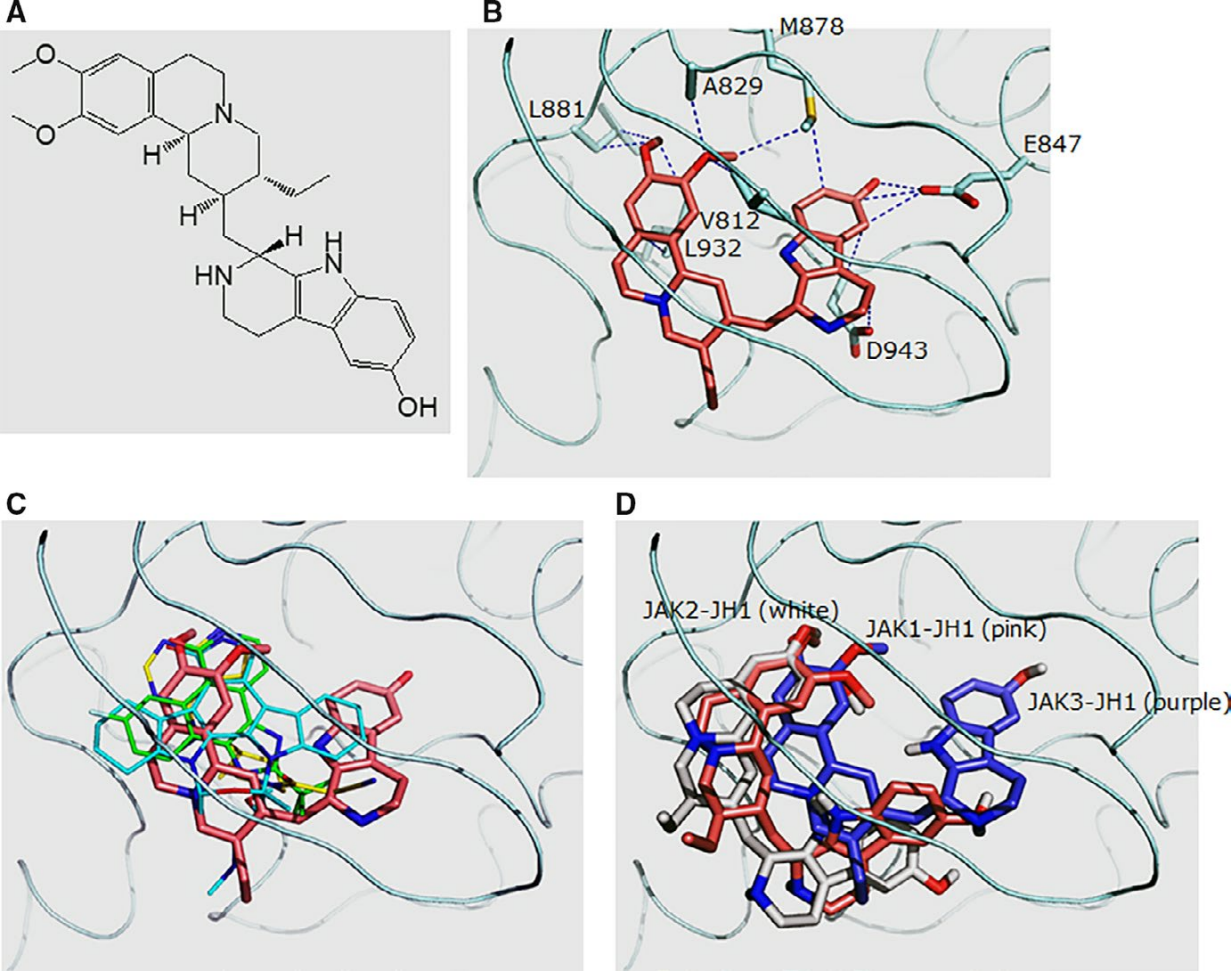
Schematic modelling of structure‐based JAK3 computational database screening. A, The chemical structure of tubulosine (C_29_H_37_N_3_O_3_). B, Predicted binding model between tubulosine and the JAK3 kinase domain (JAK3‐JH1). Tubulosine is coloured in pink. The residues that contact tubulosine with side chain atoms within 3.5 Å are labelled. C, Overlay of different ligands in complex with JAK3‐JH1. The following structures are shown: tubulosine (pink), AFN941 (cyan), CP‐690,550 (yellow) and CMP‐6 (green). These structures were generated from PDB files of 1YVJ,^24^ 3LXK^26^ and 3LXL,^26^ respectively. D, Predicted binding model between tubulosine and the kinase domains of JAK family members JAK1 (JAK1‐JH1), JAK2 (JAK2‐JH1) and JAK3 (JAK3‐JH1). JAK1‐JH1, JAK2‐JH1 and JAK3‐JH1 are coloured in pink, white and purple, respectively. All the structural figures were generated using Pymol (http://pymol.sourceforge.net/). JAK3, Janus kinase 3; JH1, Janus homology 1

Tubulosine (Figure 1A) has been deposited to the Developmental Therapeutics Program/National Cancer Institute (NCI) by the outside originators of the materials and has been available to investigators for non‐clinical research purposes. IL‐2 and prolactin (PRL) were obtained from PeproTech (Rocky Hill, NJ, USA), and interferon‐alpha (IFN‐α) was obtained from R&D Systems (Minneapolis, MN, USA). AG‐490 and ATP were purchased from Sigma‐Aldrich (St. Louis, MO, USA).

Antibodies specific for phospho‐JAK1 (#74129), JAK1 (#3332), phospho‐JAK2 (#3776), JAK2 (#3230), phospho‐JAK3 (#5031), phospho‐TYK2 (#68790), TYK2 (#14193), phospho‐STAT1 (#7167), STAT1 (#9175), phospho‐STAT3 (#9145), phospho‐STAT5 (#9359), phospho‐Src (#2101), Src (#2109), phospho‐Lyn (#2731), Lyn (#2796), phospho‐Akt (#4060), Akt (#4691), phospho‐extracellular signal‐regulated kinase (ERK)1/2 (#9101), ERK1/2 (#9102), PARP (#9452), caspase‐3 (#14220), caspase‐9 (#9502), Bcl‐2 (#2876), Bcl‐xL (#2762), Mcl‐1 (#4572), survivin (#2802), Bax (#2772), Bad (#9268), p21 (#2947), p27 (#3686), receptor‐interacting protein 3 (RIP3) (#95702), Beclin 1 (#3495), LC3B (#3868) and GAPDH (#2118) were obtained from Cell Signaling Technology (Danvers, MA, USA). Antibodies specific for JAK3 (sc‐6932), STAT3 (sc‐482) and STAT5 (sc‐835) were obtained from Santa Cruz Biotechnology (Santa Cruz, CA, USA).

### 2.2 Structure‐based computational database screening

To identify novel small molecules that target JAK3, we employed the AutoDock version 4.2^21^ and performed virtual screening using the kinase domain of JAK3 (JAK3‐Janus homology 1 [JH1], PDB ID: 1YVJ)^22^ with the NCI diversity set of compounds. The docking consists of two stages. In the first stage, the protein coordinates from the complex structure (PDB ID: 1YVJ) between the JAK3‐JH1 and its inhibitor staurosporine analog AFN941^22^ were chosen as templates. After removing the ligand and solvent molecules, AMBER software^23^ added hydrogen atoms, which was based on the PDB2PQR‐determined ionizable states in Asp, Glu, His and Lys residues.^24^ In the second step, we made detailed calculations with the decreased candidates by the initial stage. It included two JAK3‐JH1 structures complex with CP‐690,550 (PDB ID: 3LXK) and CMP‐6 (PDB ID: 3LXL) in addition.^25^ Besides, we generated 30 initial conformations of each small molecule by AMBER package and performed ensemble docking with 90 runs per compound. The resulting structures were clustered based on the structural similarity that was quantified by the root‐mean‐square deviation (RMSD) value between structures. We chose the centroid of the most populated clusters as a representative. The values of 100 and 500 000 were the parameters for the number of individuals in population (*ga_pop_size*) and the maximum number of generations (*ga_num_evals*), respectively, for the generic algorithm in AutoDock. In‐house written software and scripts automated all the procedures. To understand the common and different features of tubulosine in binding to the JAK1‐JH1 and JAK2‐JH1, we repeated the docking by using six structures from JAK1‐JH1 or JAK2‐JH1 as templates with an identical procedure. The PDB codes of the structures are 3EYG,^26^ 3EYH,^26^ 2B7A,^27^ 2W1I,^28^ 3E62^28^ and 3FUP,^26^ for JAK1‐JH1 and JAK2‐JH1, respectively.

### 2.3 Cell lines and cell culture

The human Hodgkin's lymphoma HDLM‐2 and L540 cells and B‐cell‐derived human multiple myeloma U266 cells were obtained from the German Collection of Microorganisms and Cell Cultures (DSMZ, Braunschweig, Germany). The human prostate cancer DU145 cells and epidermoid carcinoma A431 cells were obtained from the American Type Culture Collection (Manassas, VA, USA). Pre‐B leukaemia BKO‐84 cells derived from the BLNK^−/−^ mouse were obtained from Dr Daisuke Kitamura,^12^ bone marrow‐derived pro‐B cells BaF3 that stably express mutant *JAK3* (BaF3/JAK3^V674A^), TEL‐JAK2 (BaF3/TEL‐JAK2) and TEL‐JAK3 (BaF3/TEL‐JAK3) were kindly provided by Dr Hiroyuki Mano^29^ and Dr Olivier A. Bernard,^30^ and rat pre‐T lymphoma Nb2 cells were obtained from Dr Charles V. Clevenger,^31^ respectively. Cells were maintained in DMEM or RPMI medium supplemented with 10%‐20% FBS and 1% penicillin/streptomycin (HyClone, Pittsburgh, PA, USA) or in accordance with the depositor's indications.

### 2.4 In vitro kinase assays

Enzyme samples of the JAK family members were prepared from the HDLM‐2 or L540 cells by immunoprecipitation with corresponding JAK antibodies and recombinant His‐tagged STAT3α protein was purified as a substrate. The preparation and purification of the enzyme sources and recombinant His‐tagged STAT3α protein, as well as in vitro kinase assay, were previously reported.^32^ The reaction products were subjected to SDS‐PAGE and Western blotting with appropriate antibodies. Another in vitro kinase assay was performed by KinaseProfiler™ Service (Merck Millipore, Dundee, UK) as previously reported.^33^ The IC_50_ values of JAK family members were determined by Millipore's standard radiometric assay at various concentrations of tubulosine with 10 μmol/L ATP. The K_i_ and K_m_ values against JAK3 were determined at various concentrations of tubulosine and ATP. The enzyme‐specific activity (U/mg) measured in the absence of tubulosine was plotted against the ATP concentration and fitted to the Michaelis–Menten equation, allowing estimation of the K_m__(app)_ for ATP.

### 2.5 Western blot analysis

Protein samples were separated by SDS‐PAGE and transferred to nitrocellulose membrane (Pall Corp., Port Washington, NY, USA). The membrane was blocked in a blocking buffer [5% non‐fat milk in 0.1% Tween‐20 containing Tris‐buffered saline (pH 8.0)] and subsequently incubated with corresponding primary antibodies for overnight at 4°C. Blots were washed and incubated with horseradish peroxidase‐conjugated anti‐rabbit or anti‐mouse secondary antibody (Invitrogen, Waltham, MA, USA) at room temperature for 2 hours, and the signals were detected using an ECL reagent (SurModics, Eden Prairie, MN, USA).

### 2.6 Cell proliferation and viability assays

Cells (1.0 × 10^4^ cells/well) were seeded in 96‐well plates and incubated until 70%‐80% confluence. The cells were incubated for 24‐72 hours with vehicle alone, tubulosine or AG‐490, and viable cells were counted by the trypan blue exclusion assay to measure cell proliferation. Meanwhile, cell viability was measured by the absorbance at 450 nm in a microplate reader (Molecular Devices, Sunnyvale, CA, USA) after further incubation for 2‐4 hours at 37°C, followed by the addition with 10 μL EZ‐CyTox Enhanced Cell Viability Assay Reagent (Daeil Lab Service, Seoul, Korea).

### 2.7 FACS analysis

L540 cells (1.0 × 10^6^ cells/mL) were incubated for 72 hours with vehicle alone, tubulosine or AG‐490 and then stained using an APO‐BRDU kit (Phoenix Flow Systems, San Diego, CA, USA) to analyse apoptotic cell population. Cells were fixed with 70% ice‐cold ethanol, washed with PBS, incubated with RNase at 37°C for 1 hour and then stained with propidium iodide at 4°C in the dark. Annexin V was stained with Annexin V binding buffer containing fluorescein isothiocyanate (FITC) conjugated with anti‐Annexin V antibody, according to the manufacturer's instruction. Apoptotic cell population was counted with flow cytometry using BD LSRFortessa™ cell analyser (BD Biosciences, San Jose, CA, USA).

### 2.8 Statistical analysis

Results including Western blot analysis, immunoprecipitation and in vitro kinase assay were obtained from at least two independent experiments, and the representative results are shown. Data are represented as mean ± standard error of the mean. Statistical analyses were determined by GraphPad Prism software with two‐tailed Student's *t* test. A *P* value of less than 0.05 was considered statistically significant.

## 3 RESULTS

### 3.1 Identification of tubulosine as a JAK3 inhibitor

Targeting JAK3/STAT signalling is considered as a valuable therapeutic strategy to treat immune‐ and inflammation‐mediated diseases.^34^ JH1 domain is the kinase domain of JAK family proteins, which is essential for the enzymatic activity.^35^ To identify novel small molecules that inhibit the kinase activity of JAK3, we performed structure‐based computational database screening using the 3D structure of the JAK3 kinase domain (PDB ID: 1YVJ)^22^ and the compounds from the NCI diversity set. The dockings with the reference molecules AFN941, CP‐690,550 and CMP‐6^22^, ^25^ displayed encouraging performance.

We first performed the dockings with three reference molecules that have structures with a known mechanism of complexation with JAK3‐JH1. Each molecule was docked into three coordinates of JAK3‐JH1‐1YVJ,^22^ 3LXK^23^ and 3LXL.^23^ After clustering, the most populated clusters revealed the lowest energies. Representative structures from the best clusters resulted in similar conformations to those originally found in X‐ray structures within an RMSD value of 1.0 Å. The starting conformers of each molecule were produced from 2D chemical structures in the public database (http://pubchem.ncbi.nlm.nih.gov) rather than X‐ray structures. The model structure of tubulosine in complex with JAK3‐JH1 revealed contacts with the side‐chain atoms of Val‐812, Ala‐829, Glu‐847, Met‐878, Leu‐881, Leu‐932 and Asp‐943 (Figure 1B). The calculated binding free energy between JAK3‐JH1 and tubulosine was −11.79 kcal/mol. On the other hand, those of AFN941, CP‐690,550 and CMP‐6 were −10.64, −9.23 and −10.32 kcal/mol, respectively (Figure 1C), implicating that tubulosine has a higher affinity than the reference molecules for JAK3‐JH1. The binding mode shared similarities with AFN941, CP‐690,550 and CMP‐6 in the region around two –OCH_3_ moieties. However, there were also differences in the region contacting Asp‐847.

The selectivity of tubulosine towards JAK3‐JH1 over JAK1‐JH1 and JAK2‐JH1 obtained from the experimental assays is consistent with the results from in silico calculations. The binding free energies estimated with AutoDock for JAK1‐JH1 and JAK2‐JH1 were −11.10 and −10.83 kcal/mol, respectively. Both were higher than −11.79 (kcal/mol) in JAK3‐JH1, suggesting weaker bindings. The difference in the binding free energies between docked structures was mainly caused by the contacts around Asp‐847 (Figure 1D). Tubulosine closely fit into the region of JAK3‐JH1, unlike in JAK1‐JH1 and JAK2‐JH1, which in turn increased the contact area between the ligand and the protein.

### 3.2 Tubulosine inhibits JAK3 kinase activity by binding the ATP‐binding site

**FIGURE 2:**
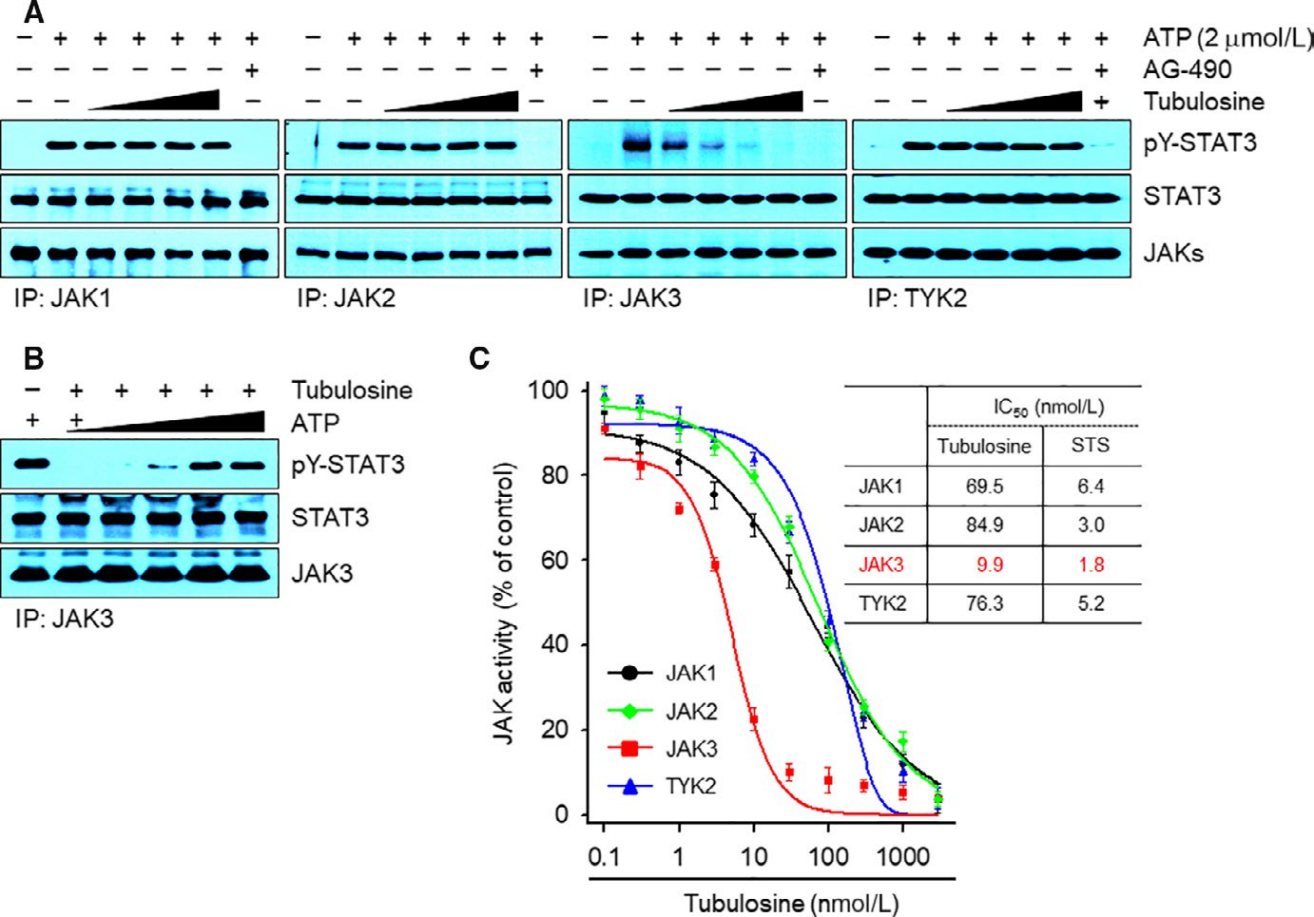
Tubulosine selectively inhibits JAK3 kinase activity in vitro. A, Each JAK immunoprecipitate was pre‐incubated with either vehicle (0.1% DMSO) alone, tubulosine (25, 50, 75 and 100 nmol/L) or the pan‐JAK inhibitor AG‐490 (150 μmol/L) for 1 h. Kinase reactions were subsequently performed by addition with His‐tagged recombinant STAT3α protein (2 μg) and ATP (2 μmol/L) for 30 min at 30°C. The reaction products were visualized by Western blot analysis using antibodies specific for the corresponding target molecules indicated. B, The in vitro JAK3 kinase assay was performed using the JAK3 immunoprecipitates incubation with increasing concentrations of ATP (2, 10, 30, 100 and 300 μmol/L) in the absence or presence of tubulosine (100 nmol/L) as described above. C, In vitro kinase assays for the JAK family members were performed using the KinaseProfiler™ Services from Merck Millipore (n = 2). The IC_50_ values of tubulosine are shown in the table and staurosporine (STS) served as a positive control. JAK3, Janus kinase 3

Because JH1 shares significant structural homology with the seven JH domains of the JAK family, we further investigated the specificity of tubulosine for JAK3‐JH1 over the JH1 of other JAKs using in vitro immunoprecipitation kinase assays. Immunoprecipitates were prepared from the lysates of HDLM‐2 or L540 cells that have constitutively active forms of JAK1, JAK2, JAK3 or TYK2.^36^ All immunoprecipitates effectively phosphorylated the tyrosine residue of STAT3α protein in the presence of ATP, indicating that the immunoprecipitates had their enzyme activities. Consistent with docking results, tubulosine effectively inhibited JAK3 kinase activity, but the kinase activities of other JAK family members were marginally affected (Figure 2A). AG‐490 also effectively blocked the kinase activities of all JAKs. Interestingly, we observed that ATP gradually decreased JAK3 kinase activity in a concentration‐dependent manner (Figure 2B, Figure S1A), indicating that tubulosine is an ATP‐competitive JAK3 inhibitor.

To determine the IC_50_ values of tubulosine for all JAKs, we performed in vitro kinase assays using the KinaseProfiler™ services. Tubulosine inhibited JAK3 kinase activity with an IC_50_ value of 9.9 nmol/L, and its activity was decreased in the presence of ATP in a concentration‐dependent manner, resulting in an EC_50_ value of 1.4 μmol/L. Although tubulosine also inhibited the kinase activities of other JAK family members, the extent of inhibition was less than that of JAK3, with IC_50_ values of 69.5, 84.9 and 76.3 nmol/L for JAK1, JAK2 and TYK2, respectively (Figure 2C, Figure S1B). We also determined K_i_ and K_m_ values for JAK3 kinase activity and found a value of 12.9 ± 1.3 nmol/L with tubulosine and 212.4 ± 25.7 μmol/L with ATP (Figure S1C). Staurosporine was used as a positive control for ATP‐competitive inhibition of JAKs. These results strongly indicate that tubulosine is a potent and selective JAK3 inhibitor.

### 3.3 Tubulosine predominantly inhibits constitutively active JAK3 signalling

**FIGURE 3:**
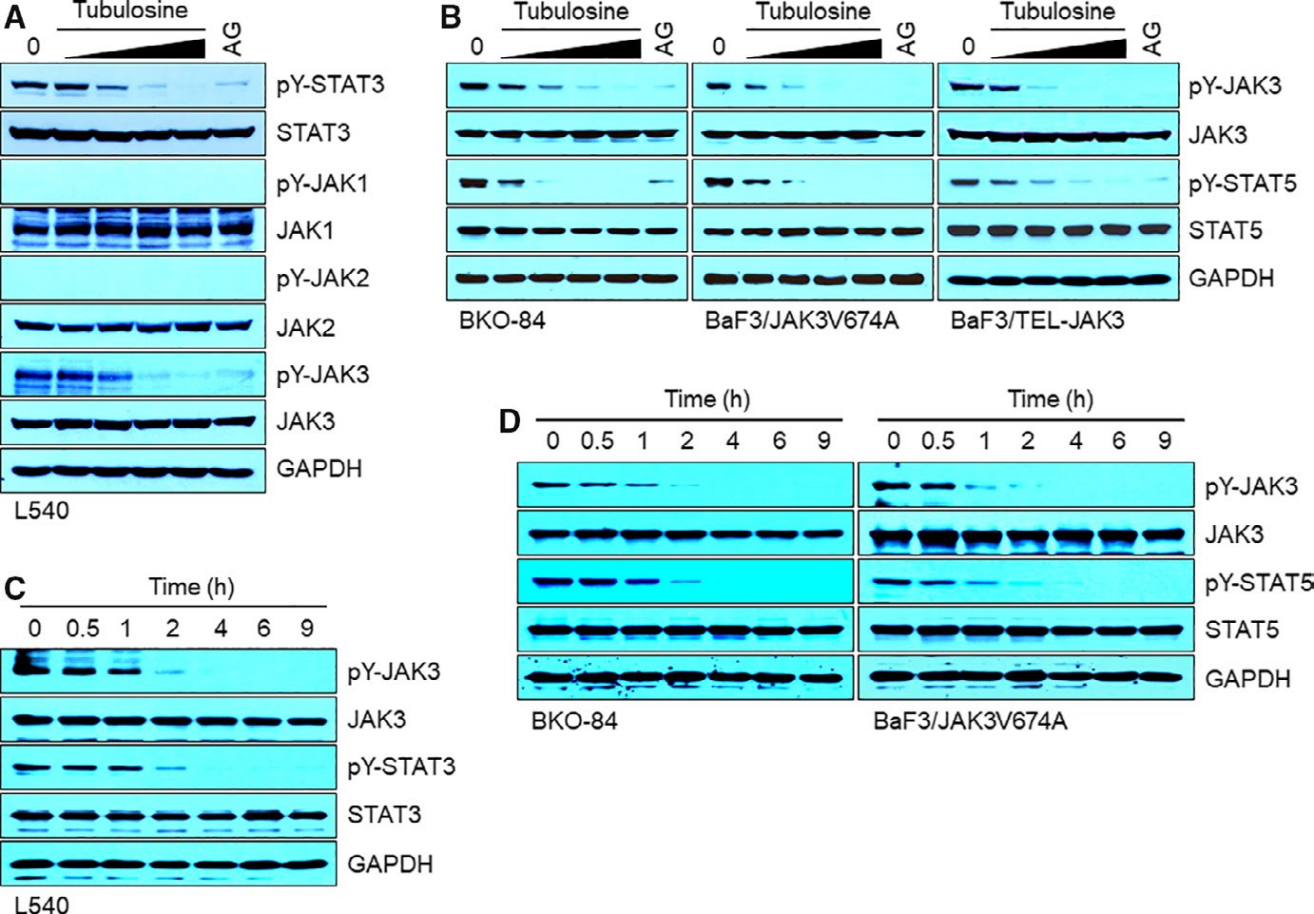
Tubulosine profoundly inhibits constitutively active JAK3/STAT signalling. A‐D, Cells were incubated for 24 h in the presence of either vehicle (0.1% DMSO) alone, tubulosine (25, 50, 75 and 100 nmol/L) or the pan‐JAK inhibitor AG‐490 (150 μmol/L). Protein samples were prepared, and Western blot analysis was performed using antibodies specific for the corresponding target molecules indicated. GAPDH served as a loading control. JAK3, Janus kinase 3

We further investigated whether tubulosine could inhibit constitutively active JAK3 signalling in various cancer cell lines. It was previously reported that the cell lines L540,^36^ BKO‐84,^12^ BaF3/JAK3^V674A29^ and BaF3/TEL‐JAK3^30^ have constitutively activated JAK3 signalling, but active forms of other JAK family members are not detectable. Tubulosine effectively inhibited the tyrosine phosphorylated forms of both JAK3 and its substrates STAT3 or STAT5 (Figure 3A,B). To gain further insight into the inhibition of JAK3 signalling, we performed time‐dependent experiment in the presence of 100 nmol/L tubulosine. The levels of tyrosine‐phosphorylated JAK3, STAT3 and STAT5 were effectively decreased by tubulosine treatment within 2 hours in all the cell lines tested (Figure 3C,D).

The cell lines HDLM‐2,^36^ DU145,^37^ A431^33^ and BaF3/TEL‐JAK2^30^ have constitutive activation forms of other JAK‐mediated signalling, but not that of JAK3. Tubulosine treatment marginally influenced tyrosine phosphorylation of JAK1, JAK2, as well as JAK1‐ and/or JAK2‐mediated STAT3 and STAT5 (Figure S2). Further, the pan‐JAK inhibitor AG‐490 profoundly inhibited the levels of tyrosine phosphorylation of all JAKs, STAT3 and STAT5 in all the cell lines tested. To determine the concentration of tubulosine that inhibited other JAK family members, HDLM‐2 and BaF3/TEL‐JAK2 cells were incubated with increasing concentrations of tubulosine ranging between 100 nmol/L and 1 μmol/L for 24 hours. A concentration of 600 nmol/L tubulosine effectively decreased the levels of tyrosine‐phosphorylated JAK1, JAK2 and TYK2 and their substrates STAT3 and STAT5 (Figure S3). These results further support that tubulosine selectively inhibits JAK3 signalling cascades versus the signalling of other JAK family members.

### 3.4 Tubulosine inhibits cytokine‐induced JAK3 signalling

**FIGURE 4:**
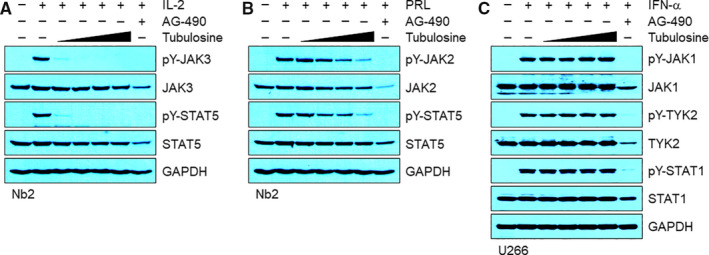
Tubulosine inhibits IL‐2‐induced JAK3/STAT5 signalling. A and B, Nb2 cells were starved for 16 h in the presence of either vehicle (0.1% DMSO) alone, tubulosine (25, 50, 75 and 100 nmol/L) or the pan‐JAK inhibitor AG‐490 (150 μmol/L). The cells were subsequently stimulated for 10 min with either IL‐2 (100 ng/mL, A) or PRL (100 ng/mL, B). C, U266 cells were incubated for 24 h in the presence of either vehicle (0.1% DMSO) alone, tubulosine (25, 50, 75 and 100 nmol/L) or the pan‐JAK inhibitor AG‐490 (150 μmol/L). The cells were subsequently stimulated for 30 min with IFN‐α (1000 U/mL). Protein samples were prepared, and Western blot analysis was performed using antibodies specific for the corresponding target molecules indicated. GAPDH served as a loading control. IFN‐α, interferon alpha; IL, interleukin;JAK3, Janus kinase 3; PRL, prolactin; STAT5, signal transducer and activator of transcription 5

We next determined the inhibitory effect of tubulosine on ligand‐mediated JAK2 and JAK3 signalling by stimulation with either IL‐2 or PRL in Nb2 cells, which have been previously used to study cytokine‐dependent activation of JAK signalling.^38^ Cells were incubated for 16 hours in the presence of vehicle alone, various concentrations of tubulosine, or AG‐490, and subsequently stimulated with either IL‐2 or PRL for 10 minutes. While phosphorylation of specific tyrosine residues on JAK2, JAK3 and STAT5 were hardly detectable in the quiescent Nb2 cells, their phosphorylation was dramatically increased by stimulation with either IL‐2 or PRL. Elevated phosphorylation levels of the JAK3 and STAT5 were almost completely disappeared after treatment with a tubulosine concentrations as low as 25 nmol/L in IL‐2‐stimulated cells (Figure 4A). However, the levels of phosphorylated JAK2 and STAT5 after PRL stimulation were only marginally decreased by tubulosine at concentrations up to 100 nmol/L (Figure 4B).

We further examined whether tubulosine could affect IFN‐α‐induced JAK1‐ and/or TYK‐2 signalling in multiple myeloma U266 cells. The levels of tyrosine‐phosphorylated JAK1, TYK2 and STAT1 were dramatically increased by stimulation with IFN‐α compared to the unstimulated cells. Tubulosine treatment did not influence the levels of their phosphorylation at concentrations up to 100 nmol/L (Figure 4C). The pan‐JAK inhibitor AG‐490 non‐selectively inhibited the tyrosine phosphorylation of all JAK and STAT family proteins in the cells stimulated with IL‐2, PRL or IFN‐α. These results strongly indicate that tubulosine has selectivity for JAK3 over other JAK family members.

### 3.5 Tubulosine does not affect other oncogenic signalling

**FIGURE 5:**
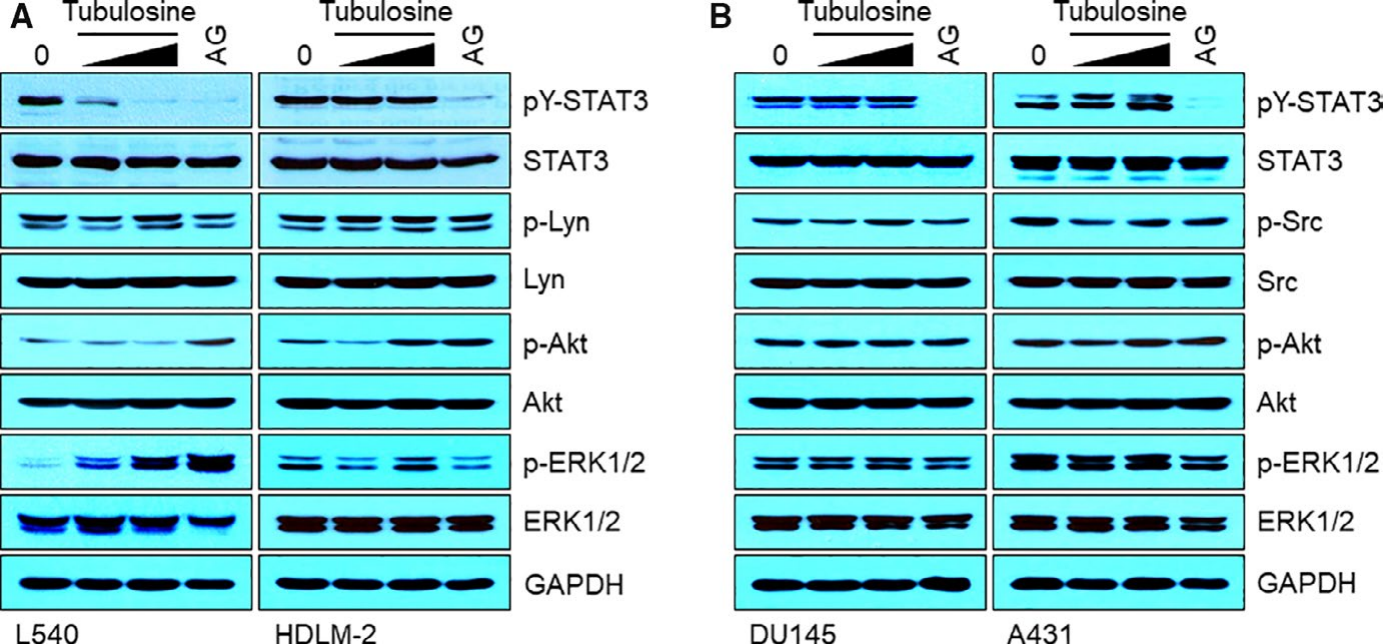
Tubulosine does not affect other oncogenic signalling. A and B, L540 and HDLM‐2 (A), and DU145 and A431 cells (B) were incubated for 24 h in the presence of either vehicle (0.1% DMSO) alone, tubulosine (50 and 100 nmol/L) or the pan‐JAK inhibitor AG‐490 (150 μmol/L). Protein samples were prepared, and Western blot analysis was performed using antibodies specific for the corresponding target molecules indicated. GAPDH served as a loading control

We further investigated whether tubulosine could affect the signalling pathways of other oncogenic components such as the Src family of non‐receptor tyrosine kinases, serine/threonine kinase Akt and ERK. Tubulosine exerted no significant inhibition on the levels of active forms of Src family tyrosine kinases, phospho‐Lyn in L540 and HDLM‐2 cells and phospho‐Src in DU145 and A431 cells (Figure 5, lanes 3, 4). We also observed no significant effect on the level of Akt phosphorylation after tubulosine treatment in the cell lines (Figure 5, lanes 5, 6). Interestingly, the phosphorylation level of ERK1/2 was dramatically increased by tubulosine treatment only in JAK3‐activated L540 cells in a concentration‐dependent manner, but not in other JAK family members‐activated HDLM‐2 cells (Figure 5, lanes 7, 8). The observation of STAT3 phosphorylation was conducted as a positive control in all tested cell lines that was inhibited by tubulosine treatment only in L540 cells, but not HDLM‐2, DU145 and A431 cells (Figure 5, lanes 1,2). Taken together, these results indicate that tubulosine selectively inhibits the JAK3 activity and subsequently results in the inhibition of its down target signal transduction, but not that of other JAK family members and oncogenic signalling components.

### 3.6 Tubulosine selectively reduces the survival and proliferation of cancer cells expressing constitutively active JAK3

**FIGURE 6:**
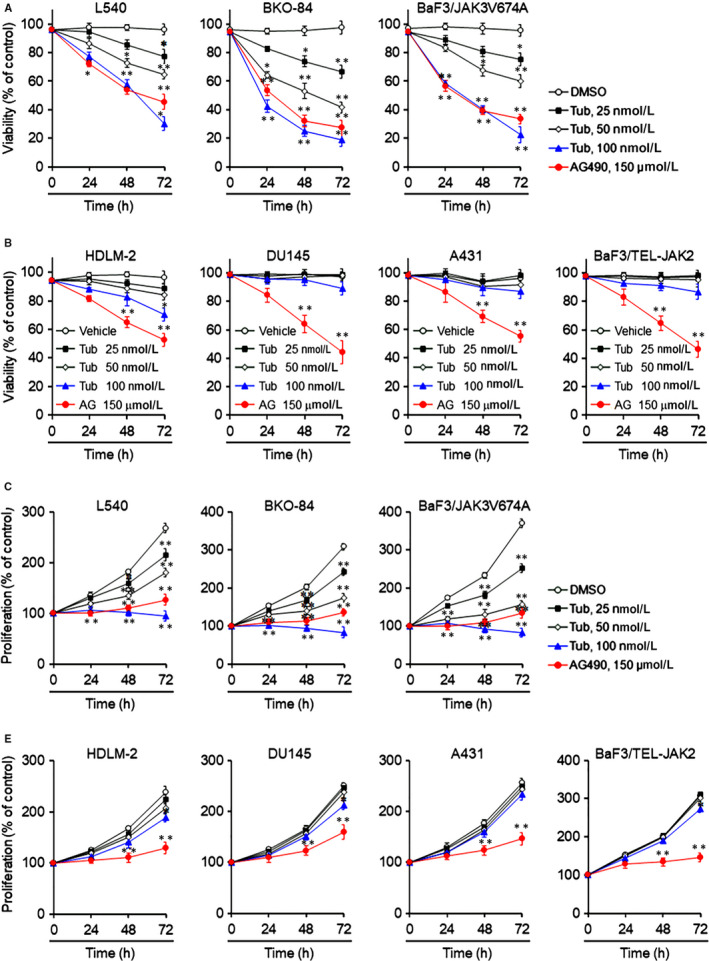
Tubulosine selectively decreases the survival and proliferation of cancer cells that constitutively express active JAK3. A and B, L540, BKO‐84 and BaF3/JAK3V674A cells (A and C) and HDLM‐2, DU145, A431 and BaF3/TEL‐JAK2 cells (B and D) were incubated with either vehicle (0.1% DMSO) alone, tubulosine (25, 50 and 100 nmol/L) or the pan‐JAK inhibitor AG‐490 (150 μmol/L) for the indicated time intervals. Cell viability and proliferation were determined using a WST‐1 reagent or by counting viable cells using the trypan blue exclusion assay. Data are represented as a % control compared to the vehicle alone‐treated group (n = 3). *P < 0.05 and **P < 0.005 compared to the vehicle‐treated group. JAK3, Janus kinase 3

Dysregulated JAK/STAT signalling leads to an increase in survival and proliferation of various types of cancer cells, suggesting that tubulosine could decrease viability only in cancer cells harbouring constitutively active JAK3 signalling. To address this possibility, we first performed a cell viability assay, followed by treatment with either vehicle alone, various concentrations of tubulosine, or AG‐490 in various cancer cells at different time intervals. As expected, tubulosine significantly reduced the viability of cancer cells that harboured constitutively activated JAK3 signalling in time‐ and concentration‐dependent manners (Figure 6A). However, treatment with tubulosine at 100 nmol/L for 72 hours only marginally affected the viability of cancer cells that harboured constitutively activated other JAK family members (Figure 6B).

The proliferation of cancer cells was also measured using the trypan blue exclusion assay. Tubulosine effectively reduced the proliferation of cancer cells that persistently expressed active JAK3 signalling in time‐ and concentration‐dependent manners (Figure 6C). In contrast, the proliferation of cancer cells with non‐JAK3 activation decreased only marginally, even at high concentrations of tubulosine treatment for 72 hours (Figure 6D). Further, the pan‐JAK inhibitor AG‐490 significantly reduced the viability and proliferation of cancer cells in all cell lines tested. These results indicate that JAK3 plays an important role in survival and proliferation of cancer cells that harbour persistent activation of JAK3 signalling. In addition, targeting JAK3 signalling by tubulosine results in decreased cell survival and proliferation, both of which events occurred coincidently.

### 3.7 Tubulosine induces apoptotic and necrotic/autophagic cell death

**FIGURE 7:**
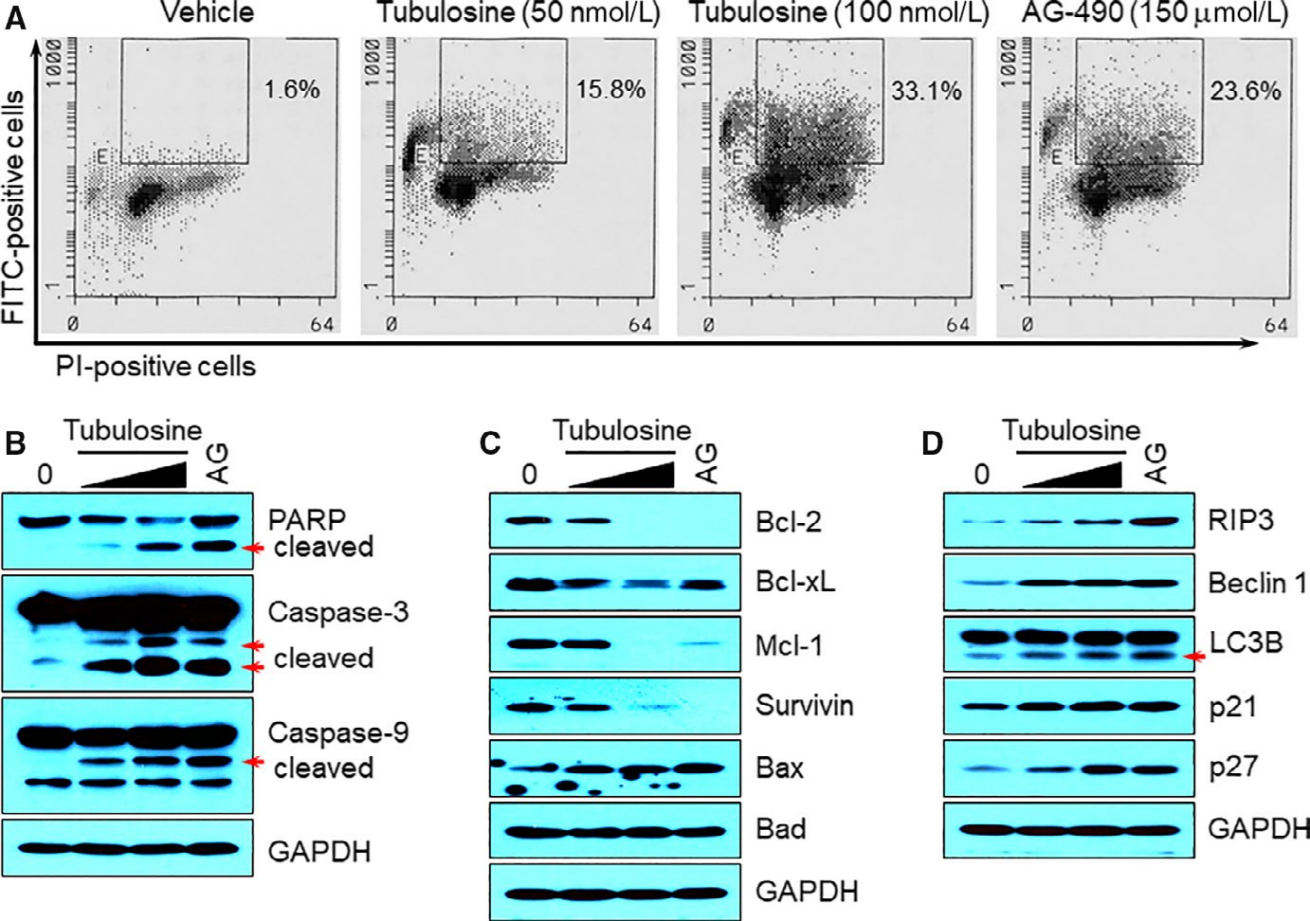
Tubulosine induces apoptotic and necrotic/autophagic cell death. A, L540 cells were incubated with either vehicle (0.1% DMSO) alone, tubulosine (50 and 100 nmol/L) or the pan‐JAK inhibitor AG‐490 (150 μmol/L) for 72 h. Cells were stained with FITC‐conjugated BrdU antibody and PI using the APO‐BRDU kit, and subsequently subjected to FACS analysis. The percentage of TUNEL‐positive cells is indicated. B‐D, L540 cells were incubated with either vehicle (0.1% DMSO) alone, tubulosine (50 and 100 nmol/L) or the pan‐JAK inhibitor AG‐490 (150 μmol/L) for 72 h. Protein samples were prepared, and Western blot analysis was performed using antibodies specific for the corresponding target molecules indicated. GAPDH served as a loading control. FITC, fluorescein isothiocyanate; PI, propidium iodide

Previous reports have demonstrated that treatment with JAK3‐specific inhibitors or siRNA induce apoptotic cell death by blocking JAK3 signalling in persistently JAK3‐activated cancer cells.^32^ Therefore, we performed TUNEL assay to determine apoptotic cell population, followed by treatment with vehicle alone, tubulosine or AG‐490 for 72 hours in L540 cells. We observed that tubulosine markedly increased apoptotic cell populations. The number of apoptotic cells increased by approximately 10‐ and 20‐fold in the presence of 50 and 100 nmol/L tubulosine compared to the vehicle‐treated control cells (Figure 7A).

To explore further the molecular mechanism by which tubulosine induced apoptotic cell death in L540 cells, we performed Western blot analysis to detect the expression levels of proteins involved in apoptosis. Tubulosine resulted in an increase in the fragmentation of the hallmark proteins of apoptosis: poly (ADP‐ribose) polymerase (PARP), caspase‐3 and caspase‐9 (Figure 7B). In addition, treatment with tubulosine resulted in suppressed the levels of anti‐apoptotic proteins such as Bcl‐2, Bcl‐xL, Mcl‐1 and survivin and also increased the level of the pro‐apoptotic protein Bax (Figure 7C). Interestingly, tubulosine treatment resulted in increasing the levels of necrotic, autophagic and/or apoptotic markers such as RIP3, Beclin‐1 and LC3B.^39^, ^40^, ^41^ It also increased the levels of cyclin‐dependent kinase inhibitors such as p21 and p27 (Figure 7D). The pan‐JAK inhibitor AG‐490 was used as a positive control, which also induced apoptotic and necrotic/autophagic cell death by affecting the expression levels of pro‐apoptotic and anti‐apoptotic proteins, and apoptotic and/or necrotic/autophagic markers. These results indicate that inhibition of JAK3 signalling by tubulosine decreases the survival and proliferation of cancer cells that have constitutive activation of JAK3 signalling by inducing apoptotic and necrotic/autophagic cell death.

## 4 DISCUSSION

The identification of active pharmacological compounds that target specific signalling pathways is a part of early‐stage drug discovery. In this study, we identified tubulosine as a potent and selective JAK3 inhibitor that predominantly targeted the enzyme activity of JAK3 kinase, as compared to other JAK family members. Tubulosine was identified by structure‐based computational database screening based on the 3D structure of the catalytic site of the JAK3 kinase domain (JAK3‐JH1, PDB ID: 1YVJ) as a biological target and the compounds from the NCI diversity set as a compound library. The selectivity of tubulosine against JAK3 kinase was demonstrated by in vitro kinase assays that effectively inhibited the intrinsic catalytic activity of JAK3 with an IC_50_ value of 9.9 nmol/L. The value was lower than that of JAK1, JAK2 and TYK2 with IC_50_ values of 69.5, 84.9 and 76.3 nmol/L, respectively, indicating that tubulosine is more selective for JAK3 than other JAK family members. In addition, inhibited the catalytic activity by tubulosine was interrupted in the presence of excess ATP with an EC_50_ value of 1.4 μmol/L and K_i_ and K_m_ values of 12.9 ± 1.3 nmol/L for JAK3 and 212.4 ± 25.7 μmol/L with ATP, indicating that this compound is an ATP‐competitive inhibitor of JAK3.

Various screening techniques have been developed to identify hit‐to‐lead compounds that show pharmacological activity. Structure‐based computational database screening is currently a popular approach in early‐stage drug discovery and the progressive optimization of lead compounds because it is faster and more cost‐efficient than traditional screening methods. It is also possible to perform large‐scale screening using chemical compound libraries. This tool is a computer‐aided method that conducts molecular docking of chemical compounds into the 3D structure of the target protein.^42^ 3D structure of target protein is generally retrieved from the PDB determined by X‐ray crystallography or NMR spectroscopy.^43^ In this study, we chose the kinase domain of JAK3 (PDB ID: 1YVJ) as the biological target and used the NCI diversity set of compounds as a library. JAK3 is a member of the Janus family of tyrosine kinases that is mainly associated with immune, inflammatory and haematopoietic disorders because they are commonly expressed in haematopoietic cells such as T cells and NK cells.^2^, ^34^, ^44^ To perform structure‐based virtual screening against JAK3, we modified the conventional docking methods by generating 30 initial conformations of each compound, performed ensemble docking and then calculated the binding energies. We compared the ligand‐binding pockets in all JAK proteins and superimposed the ligand structures onto the pockets to identify JAK3‐specifc inhibitors. Our data showed that the selectivity of tubulosine towards JAK3‐JH1 over JAK1‐JH1 and JAK2‐JH1 in the experimental assays is quantitatively consistent with the results from in silico calculations that show lower binding free energies for JAK3‐JH1 (−11.79 kcal/mol), compared to JAK1‐JH1 (−11.10 kcal/mol) and JAK2‐JH1 (−10.83 kcal/mol), respectively. This is thought to be due to the contact around Asp‐847 because it plays an essential role in increasing the contact area between tubulosine and the protein, which resulted in a tight fit into the region of JAK3‐JH1.

Various types of leukaemia and lymphoma have been recently identified from the extensive studies of human patients and mouse models that had persistent activation of JAK3 signalling with somatic mutations. In these cases, the mutations are generally associated with gain‐of‐function. On the contrary, loss‐of‐function mutations of *JAK3* in human and mouse models caused immunodeficiency such as SCID.^45^, ^46^ This evidence indicates the importance of JAK3 in the immune system and its contribution to the pathogenesis of various haematopoietic malignancies. In addition, targeting JAK3 signalling is a valuable therapeutic intervention in haematopoietic malignancies with activating alleles of *JAK3*. Recently, several JAK3 inhibitors, including CP‐690,550, VX509, ASP015K, NIBR3049 and WYE‐151650, were developed to achieve the therapeutic approaches.^2^, ^47^ These compounds inhibited the kinase activity of JAK3 with cytokine‐induced tyrosine phosphorylation of JAK3 and STAT5, as well as significantly prolonged survival in animal models for organ transplantations. Collectively, this indicates that small‐molecule inhibitors targeting JAK3 may be an effective therapeutic intervention in several cancers and immune‐related diseases caused by persistently activated JAK3 signalling.

Tubulosine is an Alangium alkaloid, which was first isolated from the bark of *Pogonopus tubulosus*.^48^ Although its isolation and structure have been known for a long time, the biological function and mechanism of action have not been clearly elucidated. The exceptions, in this regard, are its effects on amebicidal activity and inhibition of protein biosynthesis,^49^ inhibition of eukaryotic elongation factor 2‐dependent peptide chain elongation^50^ and hypoxia‐inducible factor‐1α transcriptional activity,^51^ and anti‐tumour activity.^52^, ^53^ The anti‐tumour activity was evaluated by the cytotoxicity in cancer cell lines. Recently, we reported that tubulosine inhibited JAK2/STAT3 signalling in IL‐6‐induced breast cancer.^54^ In this study, we further demonstrated a novel function of tubulosine as a JAK3 inhibitor that competes with ATP binding in the kinase domain. It selectively targeted the kinase activity of JAK3, resulting in inhibition of JAK3 activation and, therefore, suppressed the survival and proliferation of cancer cells that expressed constitutive JAK3 activation. The reduced survival and proliferation of cancer cells were associated with the increase in apoptotic and necrotic/autophagic cell death through increased the levels of cleaved pro‐apoptotic proteins and upregulation of the expression of pro‐apoptosis‐, apoptosis‐, necrosis‐ and autophagy‐related proteins. Concomitantly, tubulosine decreased the expression of anti‐apoptotic proteins and induced cell‐cycle arrest through upregulation of the expression of cyclin‐dependent kinase inhibitors. Meanwhile, tubulosine did not significantly affect the signalling of other JAK family members and other oncogenic kinases and the survival and proliferation of cancer cells lacking persistently active JAK3. Interestingly, we observed that tubulosine increased the level of ERK1/2 phosphorylation only in JAK3‐harboured cancer cells. This result may be associated with a compensation mechanism to overcome the survival and proliferation of cancer cells against the cell death caused by tubulosine.

**FIGURE 8:**
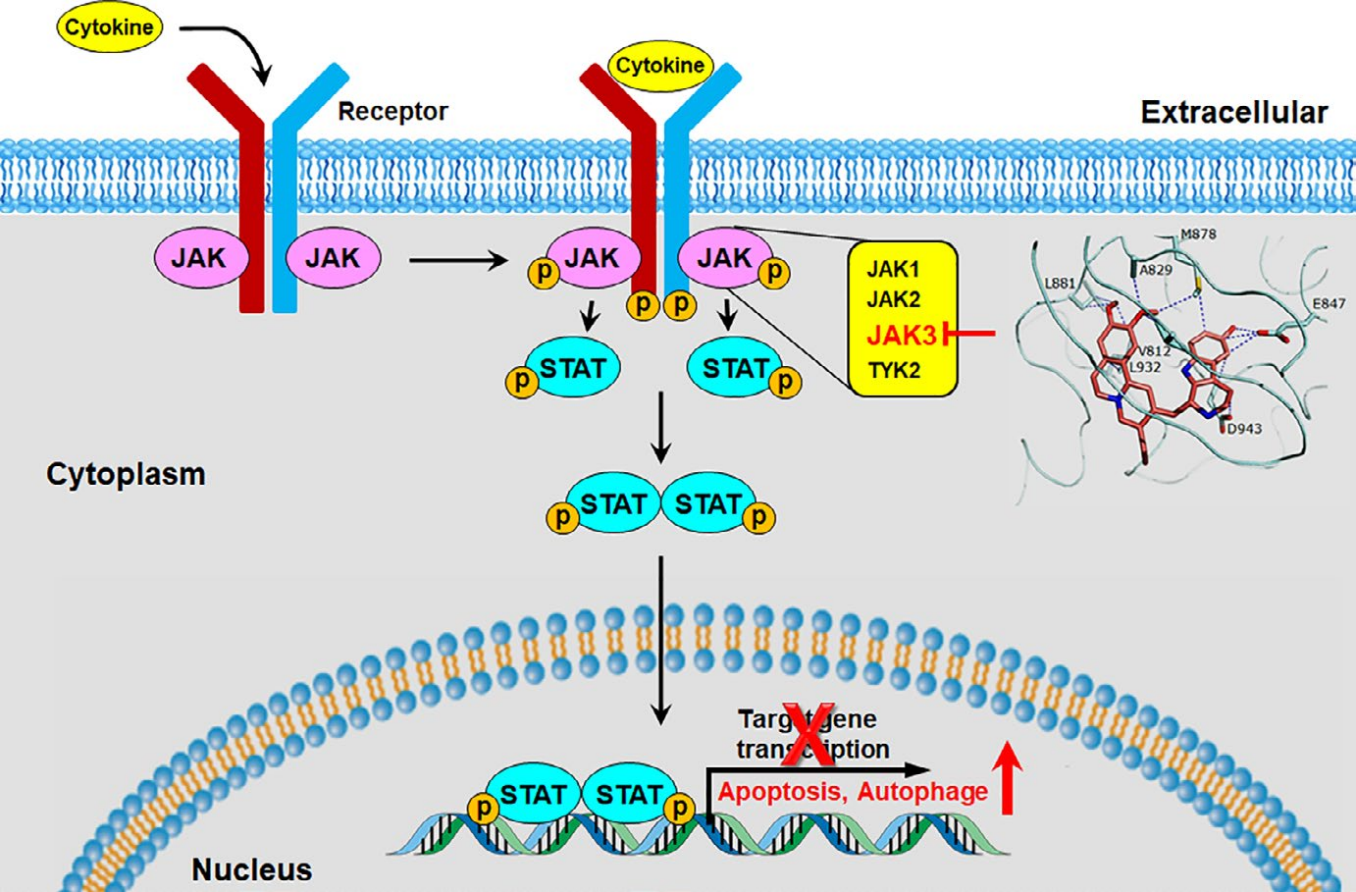
A schematic diagram illustrating the proposed action mechanism of tubulosine. Tubulosine specifically targets the signalling cascades of JAK3‐mediated STATs and their downregulated targets. JAK3, Janus kinase 3

In conclusion, we performed structure‐based computational database screening and identified tubulosine as a JAK3 inhibitor, which showed potent and moderate selectivity for JAK3 over other JAK family members. Tubulosine inhibited the catalytic activity of JAK3 and resulted in a decreased survival and proliferation of cancer cells with persistent JAK3‐activation by inducing apoptotic and necrotic/autophagic cell death (Figure 8). Our results suggest that tubulosine may have therapeutic intervention in immune‐related diseases and haematopoietic malignancies that are caused by aberrant active JAK3 signalling.

## CONFLICT OF INTEREST

The authors declare that they have no financial interest.

## AUTHORS' CONTRIBUTIONS

Byung‐Hak Kim, Eun Hee Yi, Jun‐Goo Jee, Ae Jin Jeong, Claudio Sandoval, In‐Chul Park, Gyeong Hun Baeg and Sang‐Kyu Ye designed this study. Byung‐Hak Kim and Eun Hee Yi performed experiments. Jun‐Goo Jee, Claudio Sandoval and In‐Chul Park provided experimental methods, reagents and critical suggestions. Byung‐Hak Kim, Eun Hee Yi and Ae Jin Jeong took on the statistical analysis. Byung‐Hak Kim, Eun Hee Yi, Gyeong Hun Baeg and Sang‐Kyu Ye drafted and revised the manuscript. All the authors had the approval of the submitted and published versions.

## Supporting information

Supplementary Material

Supplementary Material

## Data Availability

The data used to support findings of the study are available from the corresponding author upon request.
