# Supplementary material for: Tubulosine selectively inhibits JAK3 signalling by binding to the ATP‐binding site of the kinase of JAK3
Source: J Cell Mol Med. 2020 Jun 17;24(13):7427–38. doi: 10.1111/jcmm.15362 (PMC7339168; doi:10.1111/jcmm.15362)
Supplement: Supplementary file 1 — Supplementary Material [file JCMM-24-7427-s001.doc]

**Tubulosine selectively inhibits JAK3 signaling by binding to the ATP-binding site of the kinase of JAK3**

Byung-Hak Kim^1,2,3^, Eun Hee Yi^2,4^, Jun-Goo Jee^5^, Ae Jin Jeong^2,3^, Claudio Sandoval^1^, In-Chul Park^6^, Gyeong Hun Baeg^1,7^, and Sang-Kyu Ye^2-4,8^

^1^Department of Pediatrics, New York Medical College, Valhalla, NY, USA

^2^Department of Pharmacology and ^3^Biomedical Science Project (BK21^PLUS^), Seoul National University College of Medicine, Seoul, Republic of Korea

^4^Ischemic/Hypoxic Disease Institute, Seoul National University College of Medicine, Seoul, Republic of Korea

^5^Research Institute of Pharmaceutical Researches, College of Pharmacy, Kyungpook, National University, Daegu, Republic of Korea

^6^Division of Basic Radiation Bioscience, Korea Institute of Radiological and Medical Sciences, Seoul, Korea

^7^Department of Anatomy, Yong Loo Lin School of Medicine, National University of Singapore, Singapore, Singapore

^8^Neuro-Immune Information Storage Network Research Center, Seoul National University College of Medicine, Seoul, Republic of Korea

Byung-Hak Kim and Eun Hee Yi contributed equally to this work

***Correspondence**

Gyeong Hun Baeg, Department of Anatomy, Yong Loo Lin School of Medicine, National University of Singapore, Singapore 117594, Singapore. E-mail: [antbgh@nus.edu.sg](mailto:antbgh@nus.edu.sg)

Sang-Kyu Ye, Department of Pharmacology, Seoul National University College of Medicine, Seoul 03080, Republic of Korea. E-mail: [[sangkyu@snu.ac.kr](mailto:sangkyu@snu.ac.kr)](mailto:sangkyu@snu.ac.kr)

**SUPPORTING FIGURE LEGENDS**

**Supporting Figure S1.**


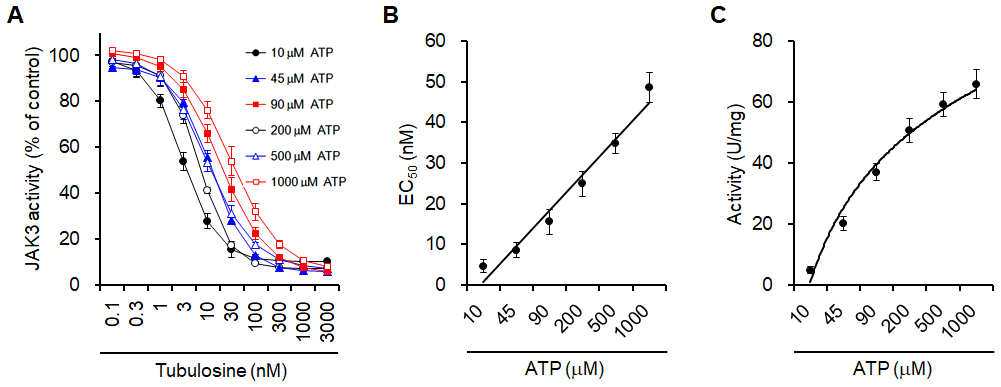


**Supporting Figure S1.** **Tubulosine** is an ATP-competitive inhibitor of JAK3 kinase. A, An *in vitro* JAK3 kinase assay was performed in an ATP concentration-dependent manner. B and C, ATP dependent EC_50_ (B) and K_m_ (C) values of tubulosine for JAK3 kinase activity are shown. All the kinase assays were conducted using the [KinaseProfiler™](http://www.biocompare.com/22662-Services/2759766-KinaseProfiler-Services/) services from Merck Millipore.

**Supporting Figure S2.**


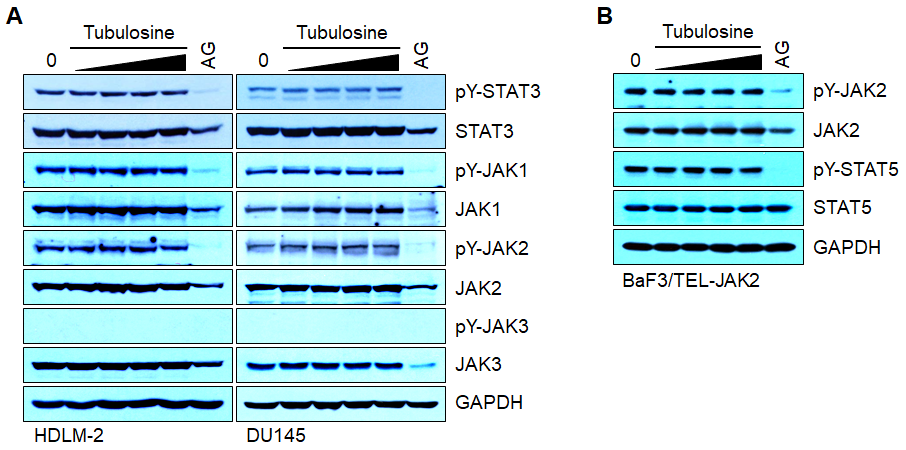


**Supporting Figure S2.** **Tubulosine does not inhibit the signaling of constitutively active other JAK family members. A and B, Cells were** incubated for 24 h in the presence of either vehicle (0.1% DMSO) alone, tubulosine (25, 50, 75, and 100 nM), or the pan-JAK inhibitor AG-490 (150 μM). Protein samples were prepared and Western blot analysis was performed using antibodies specific for the corresponding target molecules indicated. GAPDH served as a loading control.

**Supporting Figure S3.**


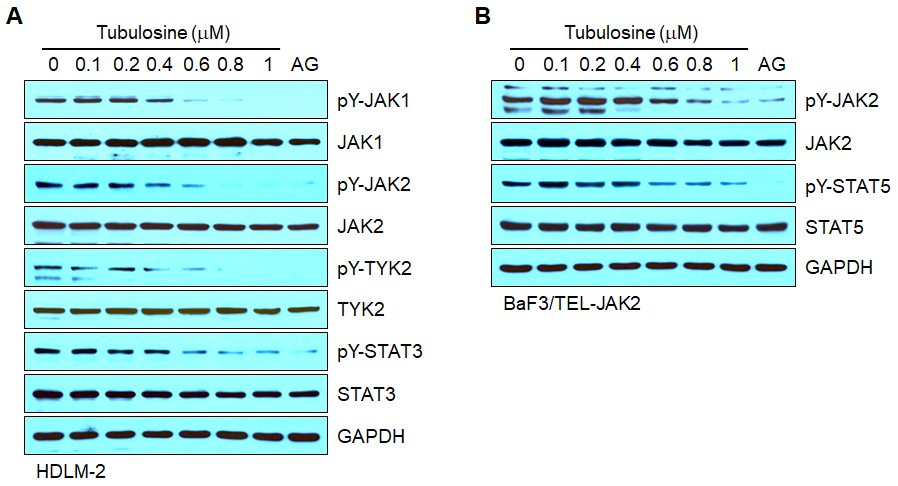


**Supporting Figure S3.** **Tubulosine inhibits the signaling of other JAK family members at high concentrations.** A and B, Cells from the human Hodgkin's lymphoma cell line HDLM-2 (A) and the murine leukemia cell line BaF3/TEL-JAK2 (B) were incubated for 24 h in the presence of either vehicle (0.1% DMSO) alone, various concentrations of tubulosine (0.1 ~ 1 μM), or the pan-JAK inhibitor AG-490 (150 μM). Protein samples were prepared and Western blot analysis was performed using antibodies specific for the corresponding target molecules indicated. GAPDH served as a loading control.
