# Supplementary material for: Tubulosine selectively inhibits JAK3 signalling by binding to the ATP‐binding site of the kinase of JAK3
Source: J Cell Mol Med. 2020 Jun 17;24(13):7427–38. doi: 10.1111/jcmm.15362 (PMC7339168; doi:10.1111/jcmm.15362)
Supplement: Supplementary file 2 — Supplementary Material [file JCMM-24-7427-s002.pptx]

## Slide 1
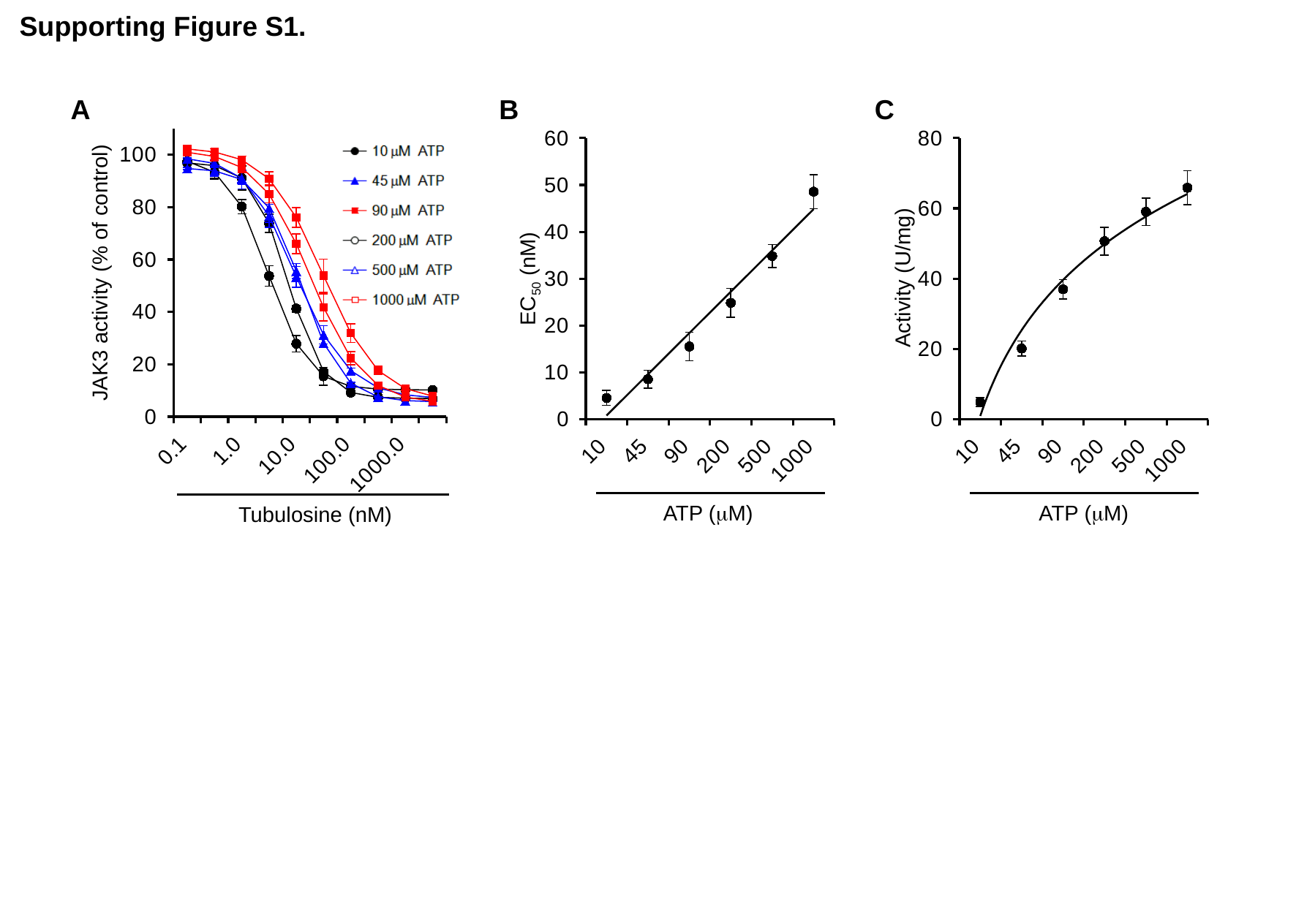

Supporting Figure S1.
A
B
C
### Chart
| Category | 10 mM | 45 mM | 90 mM | 200 mM | 500 mM | 1000 mM |
|---|---|---|---|---|---|---|
| 0.1 | 97.47264639999999 | 96.8510783999995 | 94.71412287999999 | 98.424135296 | 100.90241920000012 | 102.1804096 |
| 0.30000000000000032 | 93.32025279999995 | 95.83084799999999 | 93.82787455999967 | 96.66521203199976 | 99.31216 | 101.0349248000003 |
| 1 | 80.17399999999998 | 91.07065471999998 | 90.50105855999992 | 90.9753075199995 | 95.08240575999996 | 98.04841855999999 |
| 3 | 53.72600576000011 | 73.82349183999948 | 79.67170496 | 76.6077183999996 | 84.99440768 | 90.82298176000002 |
| 10 | 27.84642112 | 41.257526399999996 | 55.60570047999998 | 53.34699635199999 | 65.97917696 | 76.04615680000002 |
| 30 | 15.413373439999997 | 17.226251519999987 | 28.079934079999987 | 31.25403225599991 | 41.72799680000011 | 53.82260544000001 |
| 100 | 11.548785919999998 | 9.225486400000005 | 12.775732736000041 | 17.55498776320015 | 22.34351743999999 | 31.88141631999999 |
| 300 | 10.533902719999999 | 7.4073757439999985 | 7.570839488 | 11.0071707776 | 11.919522816000054 | 17.604213119999994 |
| 1000 | 10.2800512 | 7.036728000000013 | 6.137495551999973 | 8.337497241600005 | 7.5183030399999975 | 10.714908415999998 |
| 3000 | 10.217343999999999 | 6.962934015999977 | 5.767720831999979 | 7.3384073344 | 5.8587601279999975 | 7.885277696 |
### Chart
| Category | |
|---|---|
| 10 | 4.548830204357197 |
| 45 | 8.550752151214377 |
| 90 | 15.526646205357174 |
| 200 | 24.840531850178106 |
| 500 | 34.82701290475816 |
| 1000 | 48.575803981623274 |
### Chart
| Category | |
|---|---|
| 10 | 4.8658026151676985 |
| 45 | 20.117494039905363 |
| 90 | 36.984762618604414 |
| 200 | 50.693147552780054 |
| 500 | 59.03420986202255 |
| 1000 | 65.86396238131275 |
JAK3 activity (% of control)
Activity (U/mg)
EC50 (nM)
ATP (μM)
ATP (μM)
Tubulosine (nM)

## Slide 2
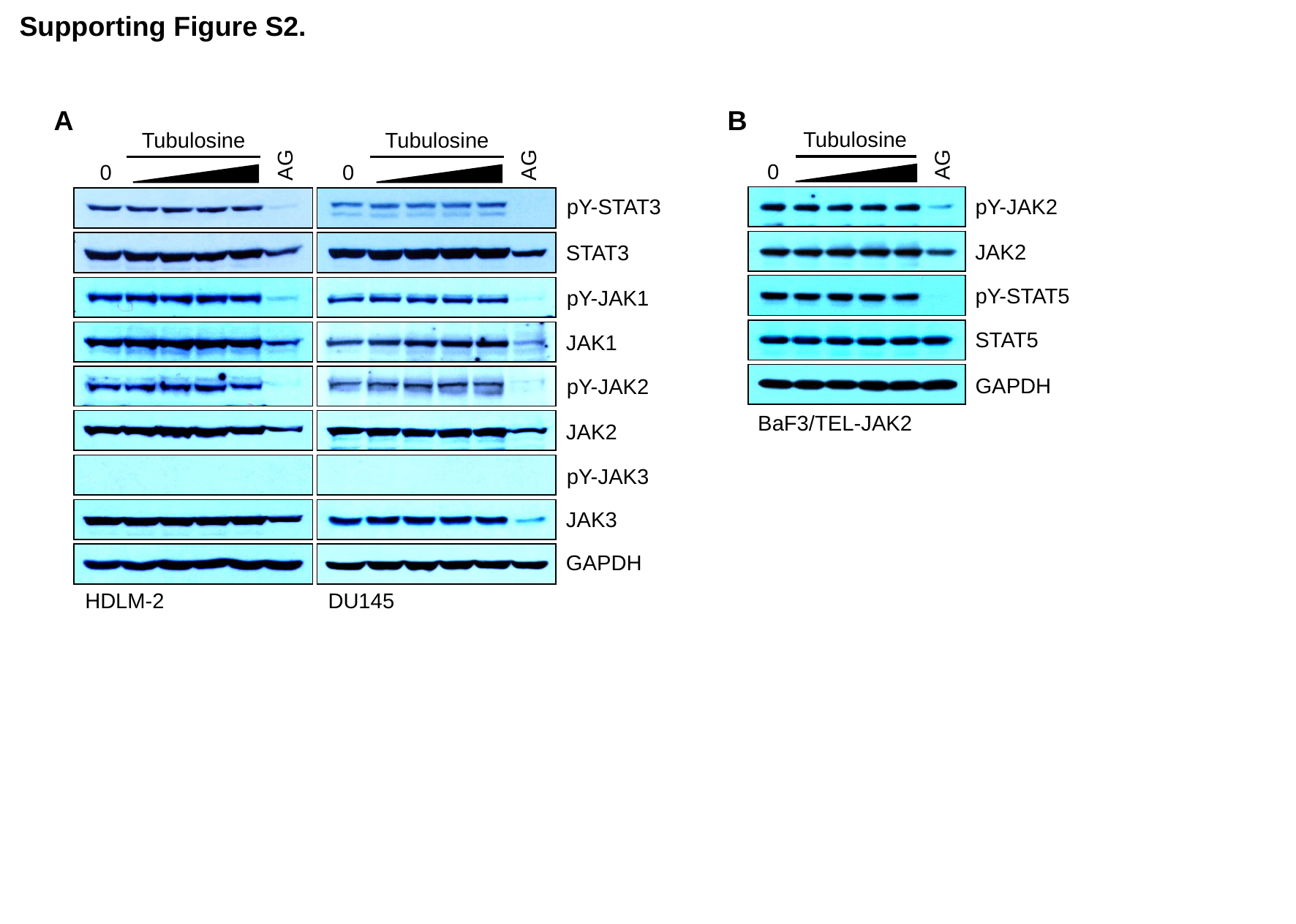

Supporting Figure S2.
A
Tubulosine
AG
0
Tubulosine
AG
0
pY-STAT3
STAT3
pY-JAK1
JAK1
pY-JAK2
JAK2
pY-JAK3
JAK3
GAPDH
HDLM-2
DU145
B
Tubulosine
AG
0
pY-JAK2
JAK2
pY-STAT5
STAT5
GAPDH
BaF3/TEL-JAK2

## Slide 3
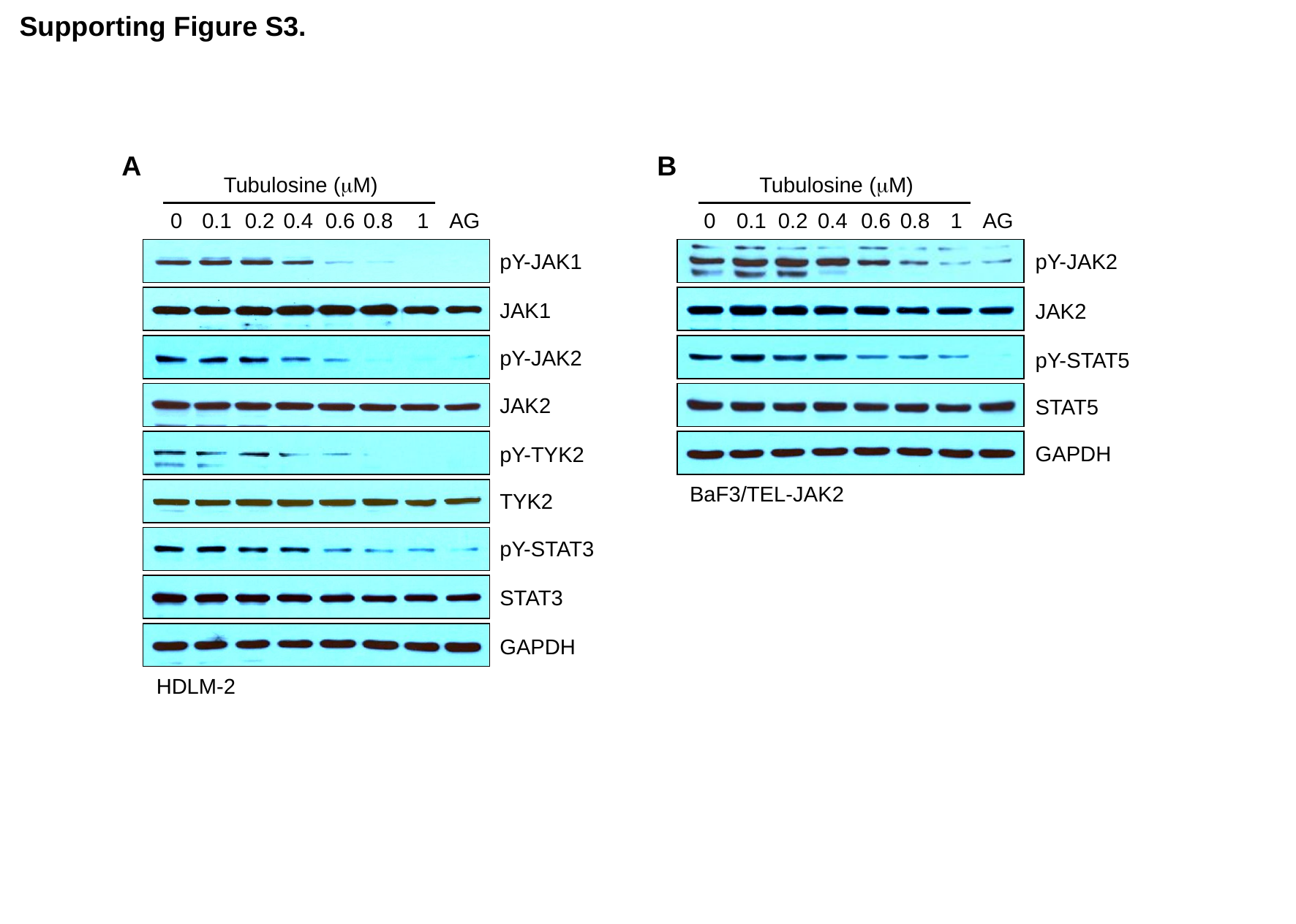

Supporting Figure S3.
A
Tubulosine (μM)
0
0.1
0.2
0.4
0.6
0.8
1
AG
pY-JAK1
JAK1
pY-JAK2
JAK2
pY-TYK2
TYK2
pY-STAT3
STAT3
GAPDH
HDLM-2
B
Tubulosine (μM)
0
0.1
0.2
0.4
0.6
0.8
1
AG
pY-JAK2
JAK2
pY-STAT5
STAT5
GAPDH
BaF3/TEL-JAK2
